# Effects of *Malania oleifera Chun* Oil on the Improvement of Learning and Memory Function in Mice

**DOI:** 10.1155/2020/8617143

**Published:** 2020-09-15

**Authors:** Rui Wu, Shaoqi Zhong, Mengmei Ni, Xuejiao Zhu, Yiyi Chen, Xuxi Chen, Lishi Zhang, Jinyao Chen

**Affiliations:** ^1^Department of Nutrition, Food Safety and Toxicology, West China School of Public Health and Healthy Food Evaluation Research Center, Food Safety Monitoring and Risk Assessment Key Laboratory of Sichuan Province, Sichuan University, Chengdu, China; ^2^West China Hospital Sichuan University, Chengdu, China

## Abstract

**Background:**

The fruits of *Malania oleifera Chun & S. K. Lee* have been highly sought after medically because its seeds have high oil content (>60%), especially the highest known proportion of nervonic acid (>55%). *Objective of the Study.* The objective was to explore the effects of different doses of *Malania oleifera Chun* oil (MOC oil) on the learning and memory of mice and to evaluate whether additional DHA algae oil and vitamin E could help MOC oil improve learning and memory and its possible mechanisms.

**Methods:**

After 30 days of oral administration of the relevant agents to mice, behavioral tests were conducted as well as detection of oxidative stress parameters (superoxide dismutase, malondialdehyde, and glutathione peroxidase) and biochemical indicators (acetylcholine, acetyl cholinesterase, and choline acetyltransferase) in the hippocampus.

**Results:**

Experimental results demonstrated that MOC oil treatment could markedly improve learning and memory of mouse models in behavioral experiments and increase the activity of GSH-PX in hippocampus and reduce the content of MDA, especially the dose of 46.27 mg/kg. The addition of DHA and VE could better assist MOC oil to improve the learning and memory, and its mechanism may be related to the inhibition of oxidative stress and restrain the activity of AChE and also increase the content of ACh.

**Conclusion:**

Our results demonstrated that MOC oil treatment could improve learning and memory impairments. Therefore, we suggest that MOC oil is a potentially important resource for the development of nervonic acid products.

## 1. Introduction

Nervonic acid (NA C24:1, Δ15), a very long-chain monounsaturated fatty acid, is a major component of mammalian brain nerve fibers and nerve cells [1, 2]. Nervonic acid can repair the damaged brain nerve and promote the regeneration of nerve cells [3]. Nervonic acid has become an effective agent for pharmaceutical and nutraceutical applications in the prevention and treatment of neurological disorders and associated diseases, including nervonic multiple sclerosis symptoms [4, 5], adrenoleukodystrophy [6], Alzheimer's disease [7, 8], and Zellweger syndrome [9]. Studies have shown that nervonic acid is essential for improving the health of children and adults with demyelination diseases diseases [4] and enhancing the neurodevelopment of formula-fed and premature babies [10, 11]. Therefore, it is recognized that nervonic acid is a bioactive supplement for human health, and the effects of which might be as important and promising as those of docosahexaenoic acid (DHA), eicosapentaenoic acid (EPA), and conjugated linoleic acid [12].

*Malania oleifera Chun* & *S. K. Lee*, a member of the family Olacaceae [13], was currently recorded on the International Union for Conservation of Nature (IUCN) Red List and listed as one of the 20 rarest plants in Yunnan Province in China. It is an evergreen broad-leaved woody tree and mainly distributed naturally in limited areas of the Karst topography of southeast Yunnan and the southwest Guangxi Provinces, China. This tree is called “garlic-fruit tree” by local residents due to its garlic-shaped fruits. *Malania oleifera Chun* oil (MOC oil), extracted from the seeds of *M. oleifera*, was also notable for its substantial phytochemical and phytopharmaceutical value [14]. It has very high content of precious fatty acids (nervonic acid (55.70%), octadecenoic acid (23.81%), docosenoic acid (13.13%), tetracosanoic acid (2.65%), dodecenoic acid (1.28%), and tricosadiynoic acid (0.99%)) [5, 15]. The application of nervonic acid to the treatment of myelin sheath diseases can reduce or prevent various mental illnesses and enhance the function of the human brain [16, 17]. Study also showed that *M. oleifera* seeds also produce glycoprotein malaria, which has high cytotoxic activity against tumor cells and is one of the most effective toxins of plant origin [5]. Historically, the seeds of *M. oleifera* have always been used to make edible oil for consumption by local people [18]. Unfortunately, *M. oleifera* are not common in the wild owing to habitat disturbances and overexploitation. Moreover, with the development of China's agriculture and planting industry, *M. oleifera* may be planted on a large scale in the future. Since MOC oil is rich in nervonic acid, it is a promising source of natural nervonic acid to promote neurohealth. However, there is no animal test study on the improvement of learning and memory function by MOC oil.

Docosahexaenoic acid (DHA) has been shown to play a key role in neuronal physiology, including neurotransmitter release regulation [19] and synaptic membrane fluidity [20]. DHA can decrease oxidative stress in rat pup brain acutely after traumatic brain injury associated with improved short-term cognitive function [21]. In addition, research shows that DHA has the effects of delaying the aging of the brain and nervous system, preventing and treating neurological diseases such as Alzheimer's disease [22] and Parkinson's disease [23]. Researchers conducted a DHA supplemental trial of children aged 7 to 9 and found that children who were supplemented with nutrition had higher reading scores and fewer behavioral problems than those who did not [24]. However, there is no consensus on DHA for enhancing brain development and improving learning and memory. Research has shown the effect of adding DHA algae oil to walnut oil, and its oil stability was measured by Schaal oven antioxidation performance test. The conclusions indicated that the addition of 15% DHA can ensure the stability of walnut oil to the greatest extent and has the longest shelf life [25].

Vitamin E (*α*-tocopherol, *α*-TOH), a group of fat-soluble vitamins with well-known antioxidant functions, has been studied in patients with moderately severe Alzheimer's disease, and was shown to be effective in slowing clinical progression [26]. Adult hippocampal neurogenesis in rats was affected by vitamin E deficiency [27]. Moreover, the addition of vitamin E to the combined formula could improve the overall stability of the oil and the antioxidant effect.

The main purpose of this study was to investigate the effect of MOC oil on the behavioral disorder of mice model and explore the optimum concentration of MOC oil and then to explore whether DHA algae oil and vitamin E can enhance the improvement of MOC oil on spatial learning and memory function in mouse models, and probe the possible underlying mechanisms, and finally, to provide reference for whether to expand the cultivation of *M. oleifera* to promote the biosynthesis of nervonic acid in the future.

## 2. Materials and Methods

### 2.1. Animals and Groups

SPF adult female Kunming mice were obtained from the Institute of Experimental, Animals Sichuan Provincial People's Hospital weighing between 26 and 30 g, housed in plastic cages individually, and kept in a regulated environment (23 ± 2°C; 55 ± 10°C% humidity) with a 12 h light/dark cycle. All experimental procedures followed the guidance of the Ethical Committee for Research on Laboratory Animals of Sichuan University and were conducted in accordance with the Guidelines for the Care and Use of Laboratory Animals approved by the European Community Guidelines. All animals were allowed 5 days to acclimatize themselves to the experimental rooms and had free access to food and water prior to experimental procedures.

The nervonic acid content of the MOC oil used in this experiment was about 46.9%. According to the recommended daily dose of NA (2.17 mg/kg) for human [28], each adult was weighed calculating 60 kg meters; then the concentration of MOC oil was at doses of 5 times, 10 times, and 20 times (23.14, 46.27, and 92.54 mg/kg) the recommended daily intake of NA here, respectively. At the first stage, for screening of the optimum concentration of MOC oil, the mice were randomly assigned to four groups (*n* = 20 per group) as follows: (1) control group (CON, i.g.): orally corn oil only; (2) low-dose group (MOC oil-L, i.g.): application of corn oil with 23.14 mg/kg MOC oil; (3) medium-dose group (MOC oil-M, i.g.): application of corn oil with 46.27 mg/kg MOC oil; and (4) high-dose group (MOC oil-H, i.g.): application of corn oil with 92.54 mg/kg MOC oil.

After the first phase of the experiment, the most effective dose of MOC oil was obtained. Based on the optimal dose, the second phase of the experiment was set up to compare the effects of different combinations of MOC oil, DHA algae oil, and vitamin E on memory of animal models. There was study on adding DHA algae oil to walnut oil, and the stability of the overall composition was determined by the Schaal oven oxidation test. It was found that the 15% DHA algae oil group had the best overall stability and the longest shelf life. Therefore, in this experiment, 85% MOC oil + 15% DHA algal oil was set.

For the second stage, to explore whether DHA algae oil and vitamin E can help MOC oil improve learning and memory of mice model. All animals were randomly divided into five groups (*n* = 20 per group) as follows: (1) control group (CON, i.g.): orally corn oil only; (2) nervonic acid group (NA, i.g.): application of corn oil with 21.67 mg/kg nervonic acid; (3) MOC oil group (MOC oil, i.g.): application of corn oil with 46.27 mg/kg MOC oil; (4) MOC oil and DHA algae oil group (MOC oil + DHA, i.g.): application of corn oil with 46.27 mg/kg MOC oil and 8.17 mg/kg DHA algae oil; and (5) MOC oil, DHA algae oil, and vitamin E group (MOC oil + DHA + VE, i.g.): application of corn oil with 46.27 mg/kg MOC oil and 8.17 mg/kg DHA algae oil and 1.00 mg/kg vitamin E.

The mice were administered with agents described above once daily on gavage and treated with standard diet and water for a month. Then, all animals would be made into memory impairment models, and their capacities for learning and memory were assessed by behavioral assessments. After the completion of behavioral evaluation, hippocampus was dissected out and appropriately treated for further analysis.

### 2.2. Reagents and Drugs

Cycloheximide (Solarbio, China) was dissolved in 0.9% saline to a concentration of 12 mg/mL. Vitamin E was purchased from Sigma-Aldrich Chemical Company, USA. Nervonic acid powder was purchased from Yuanye Biotechnology Co., Ltd. (Shanghai, China). MOC oil was provided by Kunming Kuteli Biotechnology Co., Ltd. (Yunnan, China) and stored at 4°C for further use. Malondialdehyde (MDA), acetylcholine (ACh), superoxide dismutase (SOD), glutathione peroxidase (GSH-PX), acetyl cholinesterase (AChE), and choline acetyltransferase (ChAT) levels were detected using a commercially available kit, following the manufacturer's instructions (Jiancheng Biological Technology Co., Ltd., Nanjing, China). All other chemicals were of analytical grade and obtained from Solarbio.

The nervonic acid content of the MOC oil used in this experiment was about 46.9%, and the fatty acid compositions and contents identified of MOC oil were as shown in Table 1. Since both MOC oil and DHA algae oil are rich in polyunsaturated fatty acids, they are easily oxidized and deteriorated during long-term storage, and they should be sealed at 4°C.

### 2.3. Behavioral Tests

#### 2.3.1. Step-Down Passive Avoidance Test

To assess a conditional contextual response related to learning and memory capability of animal models, the step-down passive avoidance test was a general accepted experimental method [29, 30]. This test consisted of two phases: familiar and test stage. The step-down apparatus was composed of 5 rectangular plastic dark rooms (15 × 15 × 46 cm^3^) with a stainless-steel grid floor connected to an electronic stimulant (SDT-8M, Chengdu, China). An insulation platform (4.5 cm in a diameter) was positioned in the middle of the grid floor. The memory acquisition phase was carried out by placing each mouse on the insulation platform and allowing it to free exploration in the box for 3 minutes to acclimatization. Then, the mice were firstly exposed to electric foot shock (36 V, AC) for 5 minutes. When subjected to a slight electric shock, the animal's first reaction was to jump on the insulation platform to avoid electric shock. The training was repeated for 5 consecutive days. On the 6th day, the samples were given to the stomachs, and the platform jumping training was repeated one hour later. After the training, the mice in each group (*n* = 10 per group) were injected intraperitoneally with 120 mg/kg of cycloheximide immediately to create memory consolidation disorder models [31]. The retention test was performed 1 hour after the samples were given on the 7th day. The mice were placed on the insulation platform while electrical shock was delivered. The retention latency of step-down and number of errors (times being shocked) within 3 minutes were analyzed as an index of memory function.

#### 2.3.2. Morris Water-Maze Test (MWMT)

The MWMT was conducted to evaluate the discriminative ability and spatial memory of mice model consistent with previous studies [32, 33]. The apparatus consisted of a circular pool filled with water (30 cm in depth, 23 ± 2°C) and divided into four equal quadrants (WMT-100, Chengdu, China). A fixed transparent glass platform (6 cm in diameter) was hidden 1.5 cm underneath the surface of the water in the SW quadrant (target quadrant). The test consists of two parts: positioning navigation phase and space exploration phase. The positioning navigation phase was used to measure the learning and memory acquisition abilities of experimental animals in the Morris water-maze test. Before training, animals were allowed to swim in the pool without platform for adaptation to the environment. In the training phase, all mice were initially placed on the platform to remember where they escaped, then gently released at the pseudorandom starting position, and then placed again at one of three different points. If the mouse failed to find the escaped platform within 90 s, it was placed on the platform for 10 s. Each mouse was tested four times a day and trained for five consecutive days. On the 6th day, the underwater platform was removed and entered the space exploration phase to test memory storage and reproduction capabilities of mice. Thirty minutes before the test, all animals were orally administered with 10 mL/kg 30% ethanol to establish models of memory reproduction disorder (*n* = 10 per group) [34]. All mice were allowed to freely swim in the pool with a no-platform probe trial. The escape latency to find the hidden platform and the average swimming distance in the acquisition phase, the time that the animal stayed in the circular area, and the number of crossing of the remembered-platform site in the probe trial would be recorded, respectively, by a video tracking system to be the index of spatial memory.

### 2.4. Body Weights, Relative Liver/Kidney/Spleen Indexes, Oxidative Stress Parameters, and Biochemical Indicators

The general condition and behavior of the mice were observed daily throughout the experiment. The body weights of the animals were weighed once a week, and the amounts of the agents were adjusted according to the change in body weight data. On the same day after the last behavioral assessment, the mice were immediately euthanized for cervical dislocation to obtain spleen, liver, kidney, and hippocampus for organ coefficient, oxidative stress parameters, and biochemical indicators analysis. The CA1 region of the hippocampus plays a central role in the long-term memory consolidation [35]. In the first stage, the oxidation-related parameters (the contents of MDA and the activity of SOD and GSH-PX) and biochemical indicators (the contents of ACh) in the hippocampus were measured. In the second stage of the experiment, MDA and ACh contents and SOD, GSH-PX, AChE, and ChAT activity in hippocampus of mice models were detected by different commercial assay kits according to the manufacturer's method.

### 2.5. Statistical Analysis

All analyses were performed using SPSS 26.0 software (SPSS Inc., USA) and GraphPad PRISM 7 (GraphPad Software, Inc., La Jolla, CA, USA) statistical programs. Two-sample comparisons were performed using a Student *t*-test, while multiple comparisons were made using a one-way ANOVA followed by the Student–Newman–Keuls (SNK) post hoc test. All data were presented as the means ± SEM, and statistical differences were accepted at the 5% level (*P* < 0.05), unless otherwise indicated.

## 3. Results

### 3.1. Results of the First Stage

#### 3.1.1. Body Weights and Organ Weights

At the first stage, the animals in each group developed well, and no abnormal signs and death were observed during the experiment. The results showed that there was no significant difference of weight gain and relative organ indexes of the liver, kidney, and spleen of animals among four groups as shown in Table 2 (*P* > 0.05).

#### 3.1.2. Behavioral Tests

**Figure 1:**
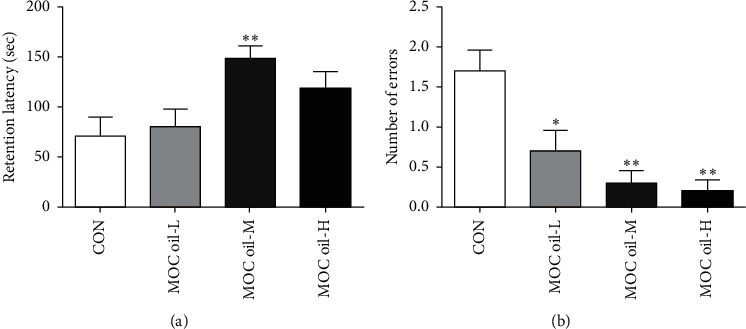
Effects of MOC oil on behaviors in the step-down passive avoidance test in cycloheximide-injected mice in the first stage. (a) Representative of the average retention latency within 3 minutes. (b) Representative of the number of errors within 3 minutes. Data are represented as mean ± S.E.M, *n* = 10 in each group. ^*∗*^, ^*∗∗*^Significantly different from those of control group at the levels of *P* < 0.05 and *P* < 0.01, respectively.

*(1) Step-Down Passive Avoidance Test*. As exhibited in Figure 1(a), compared to that of the control group, the retention latency within 3 minutes was statistically significantly increased in the MOC oil-M group (*P* < 0.01). However, retention latency in the MOC oil-L and MOC oil-H was longer in comparison with the control group but without significant difference (*P* > 0.05). As indicated in Figure 1(b), the number of mice shocked in the MOC oil-L, MOC oil-M, and MOC oil-H groups was significantly decreased than that in the control group (*P* < 0.05, *P* < 0.01, and *P* < 0.01, resp.). These findings demonstrated that MOC oil treatment could improve the impairment of memory consolidation in passive avoidance.

**Figure 2:**
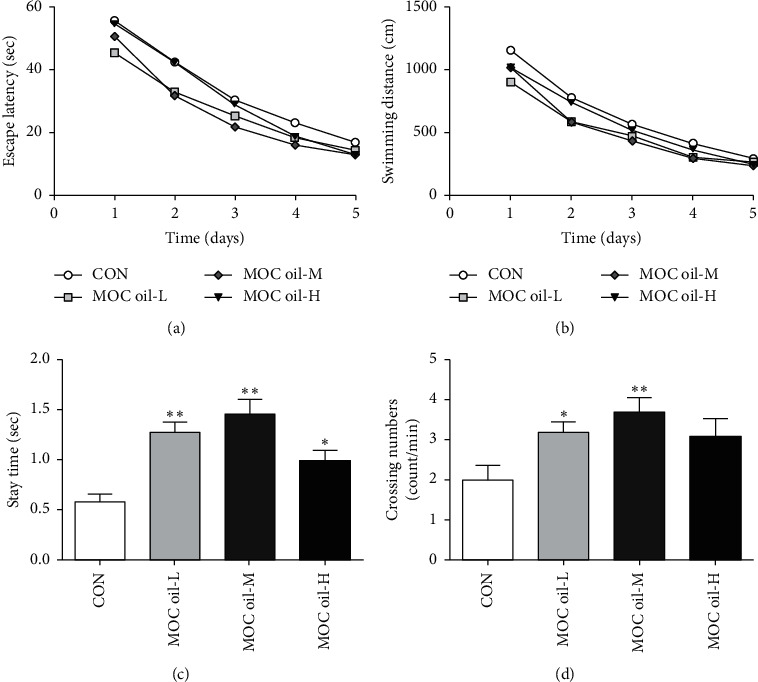
Effects of MOC oil on acquisition and retention of spatial memory mouse models by the MWMT in the first stage. (a) Escape latency to find the hidden platform in the acquisition phase. (b) Swimming distance in the acquisition phase. (c) The time that the animals stayed in the circular area in the probe trial. (d) The number of crossing of the remembered-platform site. Data were represented as mean ± SEM, *n* = 10 per group, ^*∗∗*^*P* < 0.01, ^*∗*^*P* < 0.05 versus control group.

*(2) Morris Water-Maze Test (MWMT)*. The spatial memory reproduction ability of the animals was assessed using MWMT, and the results showed that, during the 5 consecutive days of training blocks, mice who received daily doses of MOC oil-treated found the hidden platform sooner than the control ones but without statistically significant difference (*P* > 0.05, Figure 2(a)), and the escape latency of the MOC oil-M group had a slightly shorter trend than that of the MOC oil-L and MOC oil-H groups. In addition, as indicated in Figure 2(b), compared with that of the control group, the swimming distance to find the platform decreased after MOC oil administration but without significant difference among all groups (*P* > 0.05). In the probe trial, the time animals staying on the remembered-platform in the MOC oil-L, MOC oil-M, and MOC oil-H groups was significantly more than that of the control group (*P* < 0.01, *P* < 0.01, and *P* < 0.05, resp., Figure 2(c)). In addition, the results indicated that 23.14 mg/kg (*P* < 0.05) and 46.27 mg/kg MOC oil (*P* < 0.01) treatment also resulted in marked improvement of the number crossing the original platform area; moreover, 92.54 mg/kg MOC oil treatment would increase the crossing number compared to control group but the difference was not statistically significant (*P* > 0.05). These findings demonstrated that MOC oil treatment could attenuate learning and memory impairments and improve the retention of spatial memory, especially in the MOC oil-L and MOC oil-M groups.

#### 3.1.3. Oxidative Stress Parameters and Biochemical Indicators

**Figure 3:**
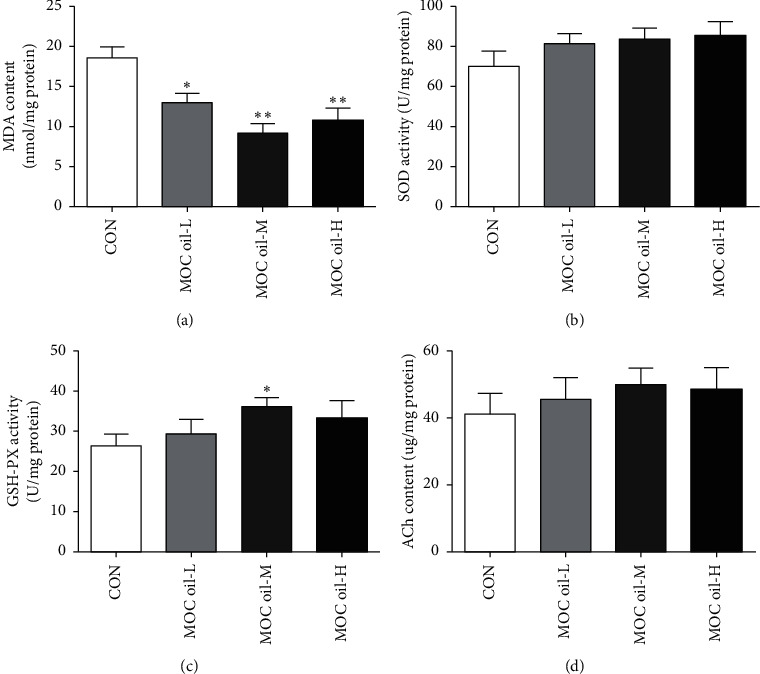
(a–d) The effects of different concentrations of MOC oil treatment on the contents of MDA and ACh and the activity of SOD and GSH-PX in the hippocampus of animal models in the first stage, respectively (mean ± SEM, *n* = 10, ^*∗*^*P* < 0.05, ^*∗∗*^*P* < 0.01 versus control group).

The levels of MDA, SOD, GSH-PX, and ACh in hippocampus were demonstrated in Figure 3. Compared to those in control group, MDA levels in MOC oil-L (*P* < 0.05), MOC oil-M (*P* < 0.01), and MOC oil-H groups (*P* < 0.01) were significantly reduced (Figure 3(a)). The activity of SOD in the hippocampus of MOC oil treatment groups showed a slight increase but without significant difference (*P* > 0.05, Figure 3(b)). GSH-PX activity in the MOC oil-M group was higher than that in the control group (*P* < 0.05, Figure 3(c)), whereas a nonsignificant enhancement was observed in low dose and high dose of MOC oil compared with that of the control group (*P* > 0.05, *P* > 0.05). ACh content in the hippocampus of MOC oil treatment groups showed a slight increase but without significant difference (*P* > 0.05, Figure 3(d)). The data of the present study could be speculated that treatment with MOC oil for 30 consecutive days could improve the oxidative stress state of the brain, especially the dose at 46.27 mg/kg.

### 3.2. Results of the Second Stage

The experimental results of the first part showed that MOC oil treatment could improve the learning and memory ability of the mouse models to some extent. Although MOC oil treatment in the MOC oil-L and MOC oil-H groups also improved the learning and memory function of the mouse model, the overall effect was not as significant as that of the MOC oil-M group, such as in the average retention latency in the step-down passive avoidance test, the stay time and crossing numbers in the Morris water-maze test, the MDA content, and the GSH-PX activity. Therefore, in the second part of the experiment, the medium dose (46.27 mg/kg) would be used as the basis for investigation to explore the effects of the formula related to MOC oil on the memory of the animal models.

#### 3.2.1. Body Weights and Organ Weights

At the second stage, body weights at sacrifice and relative liver/kidney/spleen-to-body weights of mice model in each group are summarized in Table 3. The animals in each group showed good growth and development during the experiment. No abnormal signs and death were observed. The formulation consisting of MOC oil, DHA algae oil, and vitamin E did not cause significant changes in body weight gain and relative weight of liver, kidney, and spleen (*P* > 0.05).

#### 3.2.2. Behavioral Tests

**Figure 4:**
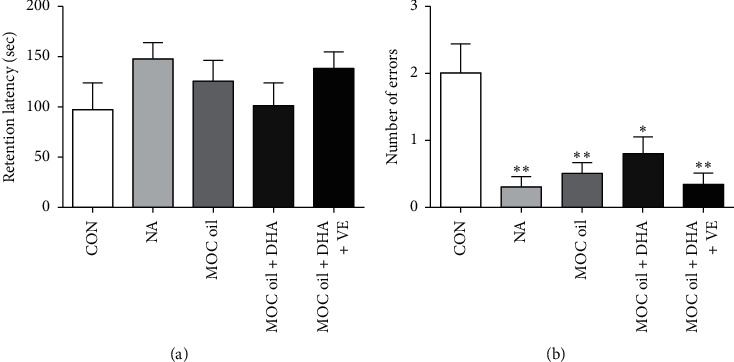
Effects of different combination of MOC oil, DHA algae oil, and vitamin E on behaviors of animal models in the step-down passive avoidance test in cycloheximide-injected mice. (a) Representative of the average retention latency within 3 minutes. (b) Representative of number of errors within 3 minutes. Data are represented as mean ± SEM, *n* = 10 per group, ^*∗∗*^*P* < 0.01, ^*∗*^*P* < 0.05 versus control group.

*(1) Step-Down Passive Avoidance Test*.  Figure 4 demonstrated the effects of different combination of MOC oil, DHA algae oil, and vitamin E on the memory function of animal models. The results showed that the retention latency in NA and MOC oil-treated mice were longer in comparison with that of the control group but without significant difference (*P* > 0.05, Figure 4(a)). The numbers of errors within 3 minutes in NA, MOC oil, MOC oil + DHA, and MOC oil + DHA + VE groups were significantly decreased than those in the control group (*P* < 0.01, *P* < 0.01, *P* < 0.05, and *P* < 0.01, resp., Figure 4(b)). In terms of the retention latency and the number of errors, the effect of MOC oil + DHA + VE combination was better than that of MOC oil alone, but without significant difference (*P* > 0.05).

**Figure 5:**
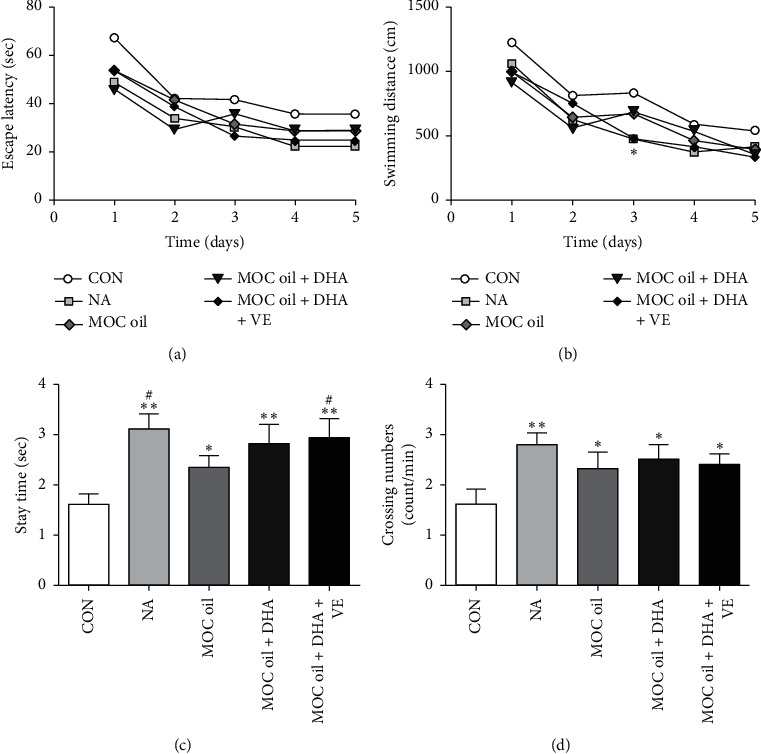
Determination of the effects of different combination of MOC oil, DHA algae oil, and vitamin E on acquisition and retention of spatial memory mouse models by the Morris water-maze test in the second stage. (a) Escape latency to find the hidden platform in the acquisition phase. (b) Swimming distance in the acquisition phase. (c) The time that the animal stayed in the circular area in the probe trial. (d) The number of crossing of the remembered-platform site. Data are represented as mean ± SEM, *n* = 10 per group, ^*∗∗*^*P* < 0.01, ^*∗*^*P* < 0.05 compared with control group, ^#^*P* < 0.05 versus MOC oil treatment.

*(2) Morris Water-Maze Test (MWMT)*. In the acquisition phase, the escape latency to reach the platform gradually decreased during the training process in all groups, whereas no significant difference was observed (Figure 5(a)). In addition, as indicated in Figure 5(b), compared with that of the control group, the swimming distance to find platform decreased after NA and MOC oil + DHA + VE administration on the third day (*P* < 0.05), which means that 21.67 mg/kg NA and 83.5% MOC oil + 14.7% DHA algae oil + 1.8% VE treatment could improve the acquisition of spatial memory. In the probe phase, compared to the control group, the time that the animals stayed in the circular area was statistically prolonged in NA and all other groups compared with that of the control group (*P* < 0.01, *P* < 0.05, *P* < 0.01, and *P* < 0.01, resp., Figure 5(c)). The number of mice crossing the platform was significantly increased in NA and all other groups in comparison with that in the control group (*P* < 0.01, *P* < 0.05, *P* < 0.05, and *P* < 0.05, resp., Figure 5(d)). It could be seen from Figure 5(c) that the effects of MOC oil + DHA and MOC oil + DHA + VE combined on the improvement of animal models memory reproduction were significantly better than that of MOC oil alone (*P* < 0.05 and *P* < 0.05, resp.), suggesting that DHA and VE might have an auxiliary effect on enhancing memory.

#### 3.2.3. Oxidative Stress Parameters and Biochemical Indicators

**Figure 6:**
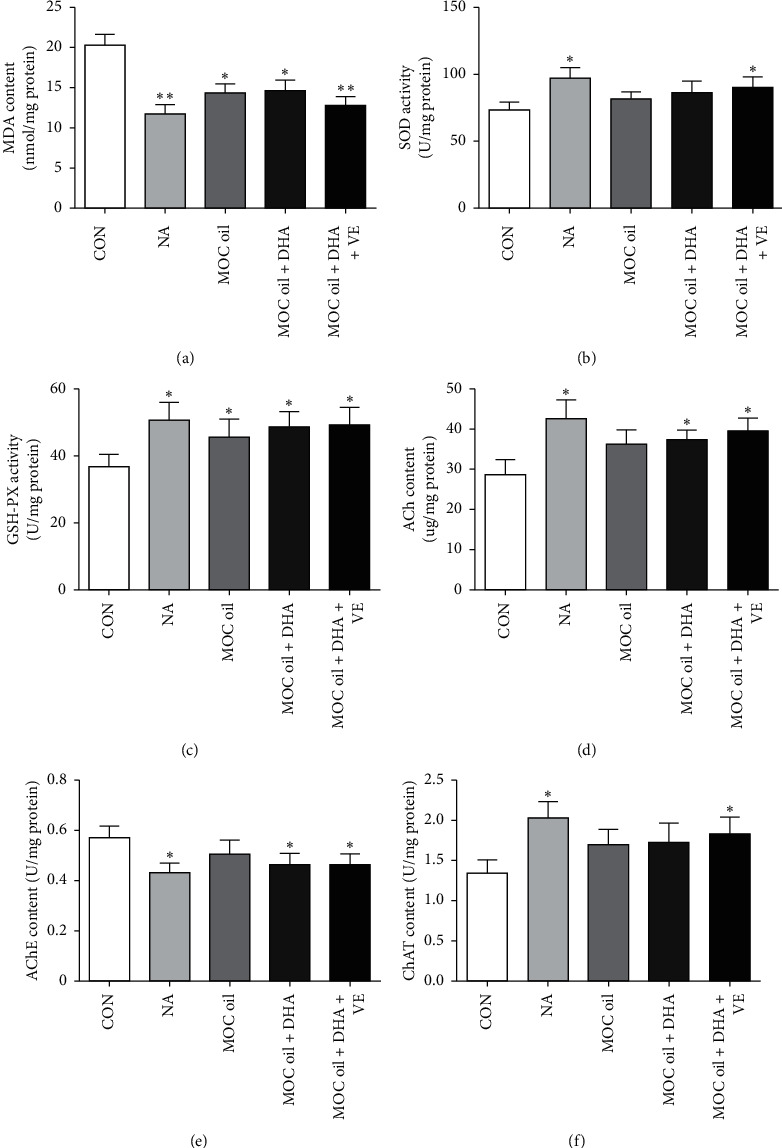
(a–f) The effects of different combination of MOC oil, DHA algae oil, and vitamin E on the contents of MDA and ACh and the activity of SOD, GSH-PX, AChE, and ChAT in the hippocampus of animal models in the second stage, respectively (mean ± SEM, *n* = 10, ^*∗∗*^*P* < 0.01, ^*∗*^*P* < 0.05 versus control group).

MDA levels in all groups exhibited significantly decreased (*P* < 0.01, *P* < 0.05, *P* < 0.05, and *P* < 0.01, resp., Figure 6(a)) compared with that in the control groups. Moreover, mice administrated with MOC oil + DHA + VE formula reduced MDA levels to a greater extent than those with pure MOC oil, but the difference was not statistically significant. Treatment with NA and MOC oil + DHA + VE resulted in an obvious enhancement in SOD activity (*P* < 0.05, *P* < 0.05, Figure 6(b)) compared to those in control group. Compared with the control group, the NA group and the combination formula of MOC oil, DHA algae oil, and VE groups exhibited a significantly increase in GSH-PX activity (*P* < 0.05, Figure 6(c)). The ACh level in the hippocampus increased significantly in the NA, MOC oil + DHA, and MOC oil + DHA + VE groups compared with the control group (*P* < 0.05, Figure 6(d)). The activity of AChE in the hippocampus of each group of animal models was shown in Figure 6(f). Compared with the control group, the AChE activity of the NA, MOC oil + DHA, and MOC oil + DHA + VE groups decreased (*P* < 0.05, *P* < 0.05, and *P* < 0.05, resp.); the AChE activity of the MOC oil group decreased, but the difference was not statistically significant (*P* > 0.05). Compared to those in control group, the ChAT activity in NA group and MOC oil + DHA + VE group was markedly increased (*P* < 0.05 and *P* < 0.05, Figure 6(f)) and in other groups showed a slight increase but without significant difference (*P* > 0.05 and *P* > 0.05, resp.). In addition, there was a slight increase in ACh content, SOD, GSH-PX, and ChAT activity in the hippocampus of the mouse models with additional DHA and VE compared with the pure MOC oil group, but the difference was not statistically significant. MDA content and AChE activity in the MOC oil + DHA and MOC oil + DHA + VE groups were lower than those in the MOC oil group but without significant difference (*P* > 0.05, Figures 6(a) and 6(e)), suggesting that the formula for adding DHA and VE might be better for the improvement of animal models memory than MOC oil alone.

## 4. Discussion

The principal findings of this study are that MOC oil containing high amounts of nervonic acid could ameliorate memory impairment of mice. The formula of 83.5% MOC oil + 14.7% DHA algae oil + 1.8% vitamin E can prevent lipid oxidation in the hippocampal tissue of mouse models, thereby greatly improving the learning and memory function of mice, which has the potential for further function products' development.

Records have shown that Zhuang residents in Guangnan County, Yunnan Province have historically taken *Malania oleifera Chun* oil [18, 36], mostly eaten by boiling hot pot or cooking, and no side effect has been reported. In the present work, we demonstrated that the intake of *Malania oleifera Chun* oil for 30 consecutive days had no adverse effects on the growth and life activities of mice, suggesting that the long-term moderate intake of MOC oil is safe.

In the first stage, the step-down passive avoidance test has been employed to assess short-term memory and discrimination capability of animal models on the basis of previous studies. Cycloheximide is a commonly used protein biosynthesis inhibitor. Previous studies have shown that intraperitoneal injection of cycloheximide will impair the memory consolidation function of animals. Therefore, all animals were injected intraperitoneally with 120 mg/kg cycloheximide 10 minutes before the test to establish memory consolidation impairment mice model [37]. The increase of the average retention latency and the decrease the number of errors in jumping on the platform were the key evidence for the improvement of animal memory ability within the specified time. The MWMT was conducted to evaluate discriminative ability and spatial memory of mice model. Ethanol is a central inhibitor, and previous studies have shown that 30% ethanol will impair memory reproduction function of mice [34, 37]. Therefore, all animals were orally administered with 10 mL/kg 30% ethanol to establish memory reproduction disorder models. During the acquisition phase, the escape latency was gradually shortened and the swimming distance to locate the platform was gradually reduced, showing that the animals were gradually gaining memory of the water maze during the training process. Compared with the control group, the mice in MOC oil-treatment groups had stronger learning ability. In the probe trial, the extended platform transit time and increased number of crossing of the removed-platform area were key evidence for improved discriminative ability and spatial memory [38, 39]. Based on the dose of 46.27 mg/kg, DHA algae oil and vitamin E were used to design different formulations. In the second part of the experiment, according to the step-down passive avoidance test, the number of errors in all groups was markedly decreased than those in the control group within 3 minutes. The increase of the time that the animals stayed in the circular area and the number that the mice crossed the remembered-platform site in all groups compared with the control group in the probe trial of MWMT were evidence of an improvement in memory reproduction.

The brain consumes an appreciable lot of oxygen and limits its antioxidant capacity; in addition, it has a large amount of polyunsaturated fatty acids and redox active transition metal ions, so it is highly susceptible to oxidative damage. Oxidative damage to synapses in the hippocampus has previously been reported to cause cognitive deficits in mice [40]. Studies have shown that memory impairment was closely related to the increase in MDA content and the decrease in SOD and GSH-PX activity [41, 42]. The level of MDA in the hippocampus can be used as a biomarker of lipid peroxidation, which is thought to be related to neuronal damage [43]. MDA is a product of lipid peroxidation and a recognized indicator of oxidative stress. It can form complexes with nucleic acids and proteins, destroy the structure of cell membranes, and thus cause cell damage [44]. GSH-PX is an important antioxidant enzyme in the body, and its content represents the strength of antioxidant capacity [45]. From the perspective of oxidative stress parameters in the mouse hippocampus, different doses of MOC oil could be varying degrees of increase the GSH-PX activity and reduce the content of MDA in the hippocampus of the mice model, of which 46.27 mg/kg MOC oil effect was best. Therefore, it is speculated that MOC oil could improve brain oxidative stress state to improve the learning and memory ability. This may be potentially related to the high levels of nervonic acid and other polyunsaturated fatty acids contained in MOC oil. Experimental results showed that all formulas could significantly reduce the MDA content and increase GSH-PX activity in the hippocampus of mice. Additionally, the SOD activity of NA and MOC oil + DHA + VE groups was significantly higher than that of control group. Moreover, the MOC oil + DHA and MOC oil + DHA + VE groups improved the lipid oxidation better than the MOC oil alone.

ACh is one of the important neurotransmitters of the central cholinergic system [46]. Its metabolism is closely related to the ability of learning and memory. It is synthesized by ChAT and decomposed by AChE [47, 48]. Therefore, the increased activity of AChE will lead to a decrease in ACh levels. From the perspective of biochemical indicators, compared with the control group, the AChE activity of NA, MOC oil + DHA, and MOC oil + DHA + VE groups was significantly reduced, while the content of ACh increased significantly. In this study, the formulation containing DHA and VE could reduce the activity of AChE and increase the activity of ChAT. It could be speculated that it can improve learning and memory by effectively regulating the level of ACh. In addition, it can be seen from the behavioral tests and oxidative stress parameters and biochemical indicators that the formulation containing DHA and VE had a stronger effect on the memory of the mouse models better than the MOC oil alone. Epidemiological and prospective cohort study has provided strong evidence to indicate that a DHA-rich diet was associated with a lowered risk of cognitive decline [49, 50]. DHA is a polyunsaturated fatty acid that is highly sensitive to oxidation reactions, especially in the antioxidant-deficient environment . Moreover, MOC oil and DHA algae oil are high in polyunsaturated fatty acids and low in vitamin E. As a key lipophilic antioxidant in humans, vitamin E plays a crucial role in protecting polyunsaturated acids associated with cell-specific membrane structures from oxidative damage [51]. Therefore, adding vitamin E to the formulation will enhance the antioxidant capacity and the stability of the entire formula.

We proposed an explanation of the mechanisms underlying the interactions of MOC oil: MOC oil could increase the activity of GSH-PX and reduce the content of MDA in the hippocampus of mice, enhance antioxidant capacity, and inhibit lipid peroxidation and oxidative stress damage effectively, thereby improving the learning and memory function.

## 5. Conclusion

In summary, we confirmed that MOC oil-treated mice showed better learning and memory function in behavioral tests. MOC oil could increase the activity GSH-PX in hippocampus and reduce the content of MDA and enhance the learning and memory function of mice. DHA algae oil and vitamin E could clearly enhance the effect of MOC oil on enhancing GSH-PX activity, reducing MDA content and reducing oxidative stress in the hippocampus of mice. At the same time, this formula could effectively reduce the activity of AChE and increase the activity of ChAT, thereby increasing the content of ACh and improving learning and memory functions, which was more efficient together than MOC oil-treated alone. Therefore, we suggest that *Malania oleifera Chun* oil is a potentially important resource for the development of neuronic acid products.

## Figures and Tables

**Table 1:** Fatty acid composition and contents of the seed oil extracted from *M. oleifera* fruits.

Fatty acids	Formula	Area (%)
Nervonic acid	C_24_H_46_O_2_	46.9
Octadecenoic acid	C_18_H_34_O_2_	30.1
Docosenoic acid	C_22_H_42_O_2_	13.9
Tetracosanoic acid	C_24_H_48_O_2_	1.9
Paullinic acid	C_20_H_38_O_2_	1.9
Linoleic acid	C_18_H_32_O_2_	1.5
Behenic acid	C_22_H_44_O_2_	1.2
Palmitic acid	C_16_H_32_O_2_	0.6
Linolenic acid	C_18_H_30_O_2_	0.6
Arachidic acid	C_20_H_40_O_2_	0.4
Stearic acid	C_18_H_36_O_2_	0.2
Others	—	0.8

**Table 2:** Body weights and relative liver/kidney/spleen weights of mice at sacrifice in the first stage (*n* = 20).

Group	Body weight gain (g)	Relative liver weight (%)	Relative kidney weight (%)	Relative spleen weight (%)
CON	8.55 ± 1.62	3.64 ± 0.38	0.94 ± 0.10	0.24 ± 0.04
MOC oil-L	9.71 ± 3.36	3.60 ± 0.21	0.99 ± 0.09	0.25 ± 0.04
MOC oil-M	9.73 ± 2.97	3.72 ± 0.25	0.98 ± 0.09	0.23 ± 0.04
MOC oil-H	9.21 ± 2.49	3.65 ± 0.27	0.99 ± 0.07	0.24 ± 0.05

**Table 3:** Body weights and relative liver/kidney/spleen weights of mice at sacrifice in the second stage (*n* = 20).

Group	Body weight gain (g)	Relative liver weight (%)	Relative kidney weight (%)	Relative spleen weight (%)
CON	14.33 ± 2.94	4.00 ± 0.43	0.90 ± 0.07	0.25 ± 0.05
NA	15.71 ± 3.30	3.80 ± 0.44	0.93 ± 0.09	0.24 ± 0.06
MOC oil	13.63 ± 2.85	3.78 ± 0.32	0.95 ± 0.09	0.29 ± 0.11
MOC oil + DHA	15.17 ± 2.44	3.88 ± 0.42	0.93 ± 0.07	0.32 ± 0.37
MOC oil + DHA + VE	14.96 ± 2.20	3.74 ± 0.48	0.93 ± 0.09	0.26 ± 0.06

## Data Availability

The data used to support the findings of this study are available from the corresponding author upon request.
